# Predictive factors for long-term survivors in advanced-stage epithelial ovarian cancer

**DOI:** 10.1016/j.xagr.2026.100675

**Published:** 2026-07-18

**Authors:** Tarinee Manchana, Nicha Assavapokee

**Affiliations:** Division of Gynecologic Oncology, Department of Obstetrics and Gynecology, Faculty of Medicine, Chulalongkorn University, Pathum Wan, Bangkok, Thailand

**Keywords:** advanced stage disease, epithelial ovarian cancer, long-term survival, prognostic factors

## Abstract

**BACKGROUND:**

Most women with epithelial ovarian cancer present with advanced-stage disease and have poor survival outcomes. However, the factor associated with long-term survival and the prognostic impact of histologic subtype in this population remain incompletely characterized.

**OBJECTIVE:**

To identify factors associated with long-term survival and to compare survival outcomes across histologic subtypes in advanced-stage epithelial ovarian cancer.

**STUDY DESIGN:**

We conducted a retrospective cohort study of 359 women with stage III to IV epithelial ovarian cancer. Multivariable logistic regression analysis was used to identify independent predictors of long-term survival, defined as overall survival of 5 years or longer. Cox proportional hazards models were applied to evaluate survival outcomes.

**RESULTS:**

Among patients with advanced-stage epithelial ovarian cancer, 109 patients (30.4%) achieved long-term survival. Median follow-up was 111.8 months. *BRCA* mutation status was independently associated with improved long-term survival (adjusted OR 8.11, 95% CI 2.46–26.67; *P*<.001). Optimal surgical cytoreduction and good performance status were also independently associated with long-term survival. In contrast, clear cell carcinoma was a negative predictor of long-term survival and associated with inferior oncologic outcomes, including a 5-year overall survival of 13.1% and an approximately twofold increased risk of mortality compared with high-grade serous carcinoma (adjusted HR 2.01, 95% CI 1.37–2.95; *P*<.001). A landmark analysis among 5-year survivors showed that the *BRCA* survival advantage did not persist beyond 5 years (*P*=.661).

**CONCLUSION:**

Long-term survival in advanced-stage epithelial ovarian cancer is independently associated with *BRCA* mutation status, optimal surgical cytoreduction, favorable performance status, and histologic subtype. Clear cell carcinoma is linked to significantly worse outcomes. These findings highlight the importance of universal genetic testing and maximal surgical cytoreduction.

**AJOG Global Reports at a Glance**

**Why was the study conducted?**

Advanced-stage epithelial ovarian cancer has poor survival, and prognostic data from Asian populations are limited.

**Key findings**

Ovarian clear cell carcinoma was a strong negative predictor of long-term survival, whereas *BRCA* mutation status and complete cytoreduction independently predicted improved outcomes.

**What does this add to what is known?**

This study identifies a disproportionate regional burden of clear cell carcinoma and supports the need for tailored treatment approaches and subtype-specific clinical trial design.

## Introduction

Epithelial ovarian, fallopian tube, and primary peritoneal carcinomas, collectively referred to as epithelial ovarian cancer, are among the most lethal gynecologic malignancies worldwide.^1^ More than 70% of affected women present with advanced disease at diagnosis, classified as International Federation of Gynecology and Obstetrics (FIGO) stage III to IV.^2^ Despite substantial advances in surgical techniques and systemic therapies, survival outcomes for epithelial ovarian cancer remain poor, with a 5-year survival rate of approximately 47% even in high-resource countries.^3^

The definition and clinical significance of long-term survival continues to evolve with therapeutic advances. While 10-year survival represents the traditional benchmark for potential cure, the 5-year timepoint remains highly relevant for several reasons: it is the standard metric for cancer registry reporting and clinical trial endpoints, it captures the period when *BRCA* mutations demonstrate their strongest survival advantage, and critically, it reflects outcomes in the modern treatment era when PARP inhibitors have fundamentally altered the natural history of *BRCA*-mutated disease.^4^^,^^5^ Factors such as histologic subtype, younger age, early-stage disease, absence of ascites, lower tumor grade, lower CA-125 levels, and favorable epidemiologic characteristics have been associated with long-term survival.^5^, ^6^, ^7^ In advanced-stage epithelial ovarian cancer, only 25% to 40% of patients with stage III disease and 15% to 20% of those with stage IV disease survive beyond 5 years.^8^ Histology plays a critical prognostic role in this setting: although high-grade serous carcinoma is the most common subtype, nonserous histologies have been linked to improved long-term and ovarian cancer–specific survival in Western populations,^9^ whereas clear-cell and mucinous carcinomas are associated with poorer outcomes in stage IV diseases.^10^

Epidemiologic studies indicate that the incidence of epithelial ovarian cancer varies by histologic subtype and race or ethnicity, with a decrease in high-grade serous cases and an increase in clear-cell and endometrioid subtypes, particularly among Asian populations.^11^ These trends suggest underlying biological and prognostic differences across geographic regions. This study was designed to evaluate clinicopathologic and surgical factors associated with long-term survival and to compare survival outcomes by histologic subtype among women with advanced-stage epithelial ovarian cancer.

## Materials and methods

### Study design and population

This retrospective cohort study enrolled all women with FIGO stage III to IV epithelial ovarian, fallopian tube, or primary peritoneal carcinoma who completed primary treatment at King Chulalongkorn Memorial Hospital between January 2009 and December 2018. Patients were de-identified through the institutional gynecologic oncology registry and electronic medical records. Among 818 women with epithelial ovarian cancer, 448 (54.8%) had stage I to II disease, and 370 (45.2%) had stage III to IV disease (Figure S1). Exclusion criteria were: (1) synchronous malignancy and (2) incomplete medical records, defined as the absence of continuous treatment records at our institution. Patients with continuous follow-up but missing individual data points for certain variables were retained in the analysis; missing values were not imputed and are specified in the corresponding table footnotes. All patient data were de-identified to ensure confidentiality. To confirm data accuracy, electronic medical records were reviewed by 2 investigators (T.M. and N.A.). Clinicopathological data and surgical outcomes were recorded. Genetic testing was offered to all patients; however, testing was performed only in patients who agreed and could afford testing.

### Study setting

The institutional treatment strategy for advanced epithelial ovarian cancer included either primary cytoreductive surgery or neoadjuvant chemotherapy followed by interval debulking surgery, as determined by the treating physicians. The standard chemotherapy regimen was intravenous carboplatin plus paclitaxel every 3 weeks, while single-agent carboplatin (AUC 5 mg/mL/min) was used when combination therapy was not feasible. Alternative regimens were administered to patients with contraindications to paclitaxel. Patients receiving neoadjuvant chemotherapy typically underwent 3 to 4 cycles, with up to 6 cycles in cases of extensive disease. After interval debulking surgery, adjuvant chemotherapy was given to complete at least 8 cycles. Residual disease was categorized into 3 groups: no residual disease (complete cytoreduction), <1 cm (optimal cytoreduction), and ≥1 cm (suboptimal cytoreduction). Disease response and progression were evaluated using clinical assessment and imaging according to the Response Evaluation Criteria in Solid Tumours (RECIST). Patients achieving complete response entered continuous surveillance, with follow-up visits every 3 months for the first 2 years, every 6 months until 5 years, and annually thereafter.

### Outcomes

Long-term survivors were defined as patients who survived ≥5 years from the date of diagnosis. Clinical and pathological characteristics were compared between long-term survival and <5-year survivors, and recurrence status was evaluated to further explore differences between groups. Progression-free survival was defined as the time from cytologic or histologic diagnosis to the date of first recurrence or last follow-up. Overall survival was defined as the time from diagnosis to death from any cause or last follow-up. December 2024 was used as the data cutoff date, with a median follow-up duration of 111.8 months (IQR 89.2–148.2).

### Statistical analysis

Categorical variables were reported as counts and percentages; continuous variables as means with standard deviations or medians with interquartile ranges. Group comparisons used chi-squared or Fisher’s exact tests for categorical data and t-tests or Mann–Whitney U tests for continuous data. Multivariable logistic regression was used to evaluate associations with long-term survival. Cox proportional hazard and Kaplan–Meier analyses were used to assess survival outcomes. All statistical analyses were performed using SPSS version 29 (IBM Corp., Armonk, NY), and 2-sided *P*-values <.05 were considered statistically significant.

## Results

A total of 359 patients with advanced epithelial ovarian cancer were included in the analysis, of whom 109 (30.4%) achieved long-term survival. The mean age at diagnosis was 56.3 years (range, 29–85 years). Most patients were diagnosed with stage III disease (76.8%), while 23.7% had stage IV disease. The 5-year overall survival rate was 32.8%, with rates of 35.1% for stage III and 25.5% for stage IV. The most common histologic subtype was high-grade serous (42.3%), followed by endometrioid carcinoma (27.6%) and clear cell carcinoma (13.1%). 65% of patients achieved optimal cytoreduction. Baseline clinical characteristics, treatments, and oncologic outcomes of patients with long-term survival are summarized in Tables 1 and 2.

**Table 1:** Clinical characteristics associated with long-term survival (N=359)

	Non-LTS (n=250)	LTS (n=109)	Total (N=359)	
	n (%)	n (%)	N (%)	*P*-value^d^
Age (y)—mean (SD)	57.0 (11.3)	55.0 (10.3)	56.3 (11.0)	.120
Body mass index^f^ (kg/m^2^)				.351
Underweight (<18.5)	32 (13.4)	14 (13.1)	46 (13.3)	
Normal weight (18.5–22.9)	96 (40.3)	52 (48.6)	148 (42.9)	
Overweight (23.0–27.4)	82 (34.5)	27 (25.2)	109 (31.6)	
Obese (≥27.5)	28 (11.8)	14 (13.1)	42 (12.2)	
Have children				.112
No	99 (39.6)	53 (48.6)	152 (42.3)	
Yes	151 (60.4)	56 (51.4)	207 (57.7)	
Family history of cancer^a^				<.001
No	222 (90.2)	80 (74.8)	302 (85.6)	
Yes	24 (9.8)	27 (25.2)	51 (14.4)	
ECOG				.004
0–1	189 (75.6)	97 (89.0)	286 (79.7)	
≥2	61 (24.4)	12 (11.0)	73 (20.3)	
Presence of ascites^b^				.203
No	67 (28.9)	37 (35.9)	104 (31.0)	
Yes	165 (71.1)	66 (64.1)	231 (69.0)	
Baseline serum CA 125 (IU/mL)^c^ – Median (IQR)	785.5 (290.5–2096.5)	1091 (331.5–3763.5)	897.0 (304.5–2402.8)	.059^e^
*BRCA* status				<.001
No mutation	41 (16.4)	24 (22.0)	65 (18.1)	
*BRCA* 1/2	5 (2.0)	17 (15.6)	22 (6.1)	
Unknown/Not performed	204 (81.6)	68 (62.4)	272 (75.8)	
Primary origin				.290
Ovary or fallopian tube	213 (85.2)	88 (80.7)	301 (83.8)	
Primary peritoneum	37 (14.8)	21 (19.3)	58 (16.2)	
FIGO stage				.117
III	185 (74.0)	89 (81.7)	274 (76.3)	
IV	65 (26.0)	20 (18.3)	85 (23.7)	
Histology				.029
High-grade serous	100 (40.0)	52 (47.7)	152 (42.3)	
Endometrioid	65 (26.0)	34 (31.2)	99 (27.6)	
Clear cell	41 (16.4)	6 (5.5)	47 (13.1)	
Low-grade serous	6 (2.4)	6 (5.5)	12 (3.3)	
Mucinous	13 (5.2)	3 (2.8)	16 (4.5)	
Others^g^	25 (10.0)	8 (7.3)	33 (9.2)	
Histologic grading				.046
Grade 1	20 (8.0)	18 (16.5)	38 (10.6)	
Grade 2	14 (5.6)	10 (9.2)	24 (6.7)	
Grade 3	172 (68.8)	65 (59.6)	237 (66.0)	
Unknown grade	44 (17.6)	16 (14.7)	60 (16.7)	

*ECOG*, Eastern Cooperative Oncology Group; *FIGO*, International Federation of Gynaecology and Obstetrics; *IQR*, interquartile range; *LTS*, long-term survivors.

Missing data footnotes:

a Family history of cancer: data unavailable for 6 patients (1.7%); percentages based on n=353

b Presence of ascites: data unavailable for 24 patients (6.7%); percentages based on n=335

c Baseline serum CA125: data unavailable for 42 patients (11.7%); reported as median (IQR) based on n=317

d Chi-squared test and Fisher’s Exact test

e Mann–Whitney U test

f According to the Asian BMI category (World Health Organization. Lancet 2004); data unavailable for 14 patients (3.9%); percentages based on n=345

g Others histology: including mixed-subtype, adenocarcinoma.

**Table 2:** Treatment and oncologic outcomes of advanced epithelial ovarian cancer (N=359)

	non-LTS (n=250)	LTS (n=109)	Total (N=359)	
	n (%)	n (%)	N (%)	*P*-value^b^
Type of surgery				.093
Primary cytoreduction	131 (52.4)	66 (60.6)	197 (54.9)	
Interval debulking surgery	94 (37.6)	39 (35.8)	133 (37.0)	
No surgery	25 (10.0)	4 (3.6)	29 (8.1)	
Residual disease (n=330)				<.001
No residual disease	78 (34.7)	57 (54.3)	135 (41.0)	
Residual <1 cm.	54 (24.0)	27 (25.7)	81 (24.5)	
Residual ≥1 cm.	93 (41.3)	21 (20.0)	114 (34.5)	
Regimen of chemotherapy^c^				.015
Carboplatin with paclitaxel	192 (79.3)	100 (91.7)	292 (83.2)	
Single carboplatin	35 (14.5)	7 (6.4)	42 (12.0)	
Others with carboplatin	15 (6.2)	2 (1.8)	17 (4.8)	
Total chemo cycles—median (range)	6 (0–18)	6 (3–18)	6 (0–18)	.042^d^
Receiving bevacizumab	5 (2.0)	4 (3.7)	9 (2.5)	.463
Receiving PARP inhibitors	-	2 (1.8)	2 (0.6)	.092
Recurrent disease	223 (89.2)	64 (58.7)	287 (79.9)	<.001
Platinum-sensitive	142 (63.7)	60 (93.8)	202 (70.4)	
Platinum-resistant	60 (26.9)	2 (3.1)	62 (21.6)	
Platinum-refractory	21 (9.4)	2 (3.1)	23 (8.0)	
Site of recurrence^a^				.054
Pelvis	33 (15.6)	7 (10.9)	40 (14.5)	
Extrapelvis	59 (27.8)	28 (43.8)	87 (31.5)	
Both	120 (56.6)	29 (45.3)	149 (54.0)	
Median PFS (mo)	11.8	57.2	16.2	<.001
Median OS (mo)	23.0	NR	35.6	<.001
Death	236 (94.4)	46 (42.2)	282 (78.6)	.758
Cancer-related death	219 (92.8)	42 (91.3)	261 (92.6)	
Non cancer-related death	17 (7.2)	4 (8.7)	21 (7.4)	

*LTS*, long-term survivors; *OS*, overall survival; *PARP*, Poly(ADP-ribose) polymerase inhibitors; *PFS*, progession-free survival.

Missing data footnotes:

a Site of recurrence: data unavailable for 11 patients with recurrent disease; percentages based on n=277

b Chi-squared test and Fisher’s Exact test

c Mann–Whitney U test

d Others with carboplatin: gemcitabine or liposomal doxorubicin or docetaxel; 8 (2.2%) did not received systemic therapy.

Long-term survivors more frequently had a family history of cancer and good performance status. Histologic subtype differed significantly between long-term survival and nonlong-term survival patients (*P*=.029), with clear cell carcinoma being more prevalent in the nonlong-term survival group (16.4% vs 5.5%). Only one-quarter of patients agreed to underwent genetic testing, and pathogenic *BRCA1*/2 variants were significantly more common among long-term survival (15.6% vs 2.0%, *P*<.001). No significant differences were observed between groups with respect to body mass index, parity, presence of ascites, baseline CA-125 level, or disease stage. Patients with long-term survival were more likely to have low-grade histology, achieve complete and optimal cytoreduction, and receive combination platinum-based chemotherapy, with all differences statistically significant. Despite advanced disease, access to targeted therapies was limited: only 2.5% of patients received bevacizumab, and only 9% of *BRCA* mutation carriers received a PARP inhibitor. Most patients (79.9%) experienced disease recurrence. Median progression-free survival was substantially longer among long-term survival compared with nonlong-term survival patients (57.2 vs 11.8 months), and long-term survival patients were more likely to have platinum-sensitive disease. Median overall survival for the entire cohort was 35.6 months, and the majority of deaths were cancer-related. Among long-term survivors, histologic grade was the only variable that differed significantly according to recurrence status (Table S1).

Multivariable logistic regression identified several independent predictors of long-term survival (Table 3). *BRCA* mutation status emerged as the strongest positive predictor, with carriers having approximately 9-fold higher odds of achieving 5-year survival compared with noncarriers (adjusted OR 8.11, 95% CI 2.46–26.67, *P*<.001). Patients who did not undergo genetic testing had similar odds of long-term survival compared with confirmed noncarriers (adjusted OR 0.70, 95% CI 0.37–1.32, *P*=.272). To evaluate whether the survival advantage associated with *BRCA* mutation persisted beyound 5 years, a landmark analysis was performed among 5-year survivors with known *BRCA* status; no statistically significant difference in survival was observed between groups (*P*=.661; Figure S2). In contrast, clear cell carcinoma was a negative predictor, associated with significantly lower odds of long-term survival compared with high-grade serous carcinoma (adjusted OR 0.33, 95% CI 0.12–0.87, *P*=.025). Suboptimal cytoreductive surgery and poor performance status were also independently associated with lower long-term survival. In a sensitivity analysis restricted to patients with known *BRCA* status, *BRCA* mutation remained independently associated with long-term survival (adjusted OR 7.26, 95% CI 2.14–24.62, *P*=.001; Table S2).

**Table 3:** Logistic regression analysis of factors associated with 5-year long-term survival

	Univariable analysis		Multivariable analysis	
	Odds ratio (95% CI)	*P*-value	Adjusted OR (95% CI)	*P*-value
Age				
<70 y	1 (reference)	.137		
≥70 y	0.60 (0.30–1.18)			
ECOG		.005		.083
0–1	1 (reference)		1 (reference)	
≥2	0.38 (0.20–0.75)		0.50 (0.23–1.09)	
BRCA status		<.001		
No mutation	1 (reference)		1 (reference)	<.001
BRCA mutation	9.05 (3.25–25.25)		8.11 (2.46–26.67)	<.001
Not tested			0.70 (0.37–1.32)	.272
Presence of ascites		.322		
No	1 (reference)			
Yes	0.79 (0.50–1.26)			
Primary origin		.292		
Ovary or fallopian tubes	1 (reference)			
Primary peritoneum	1.37 (0.76–2.48)			
Stage		.119		
III	1 (reference)			
IV	0.64 (0.37–1.12)			
Histology				
High-grade serous	1 (reference)	.042	1 (reference)	.023
Endometrioid	1.01 (0.59–1.72)	.983	1.59 (0.87–2.91)	.133
Clear cell	0.28 (0.11–0.71)	.007	0.33 (0.12–0.87)	.025
Others	0.74 (0.39–1.43)	.372	0.95 (0.48–2.11)	.906
Residual disease				
No residual disease	1 (reference)	<.001	1 (reference)	<.001
Residual <1 cm.	0.68 (0.39–1.22)	.195	0.68 (0.37–1.26)	.223
Residual ≥1 cm.	0.31 (0.17–0.55)	<.001	0.28 (0.15–0.53)	<.001
Regimen of chemotherapy				.386
Carboplatin with paclitaxel	1 (reference)	.045	1 (reference)	
Single carboplatin	0.42 (0.18–0.98)		0.65 (0.24–1.72)	

**Figure 1:**
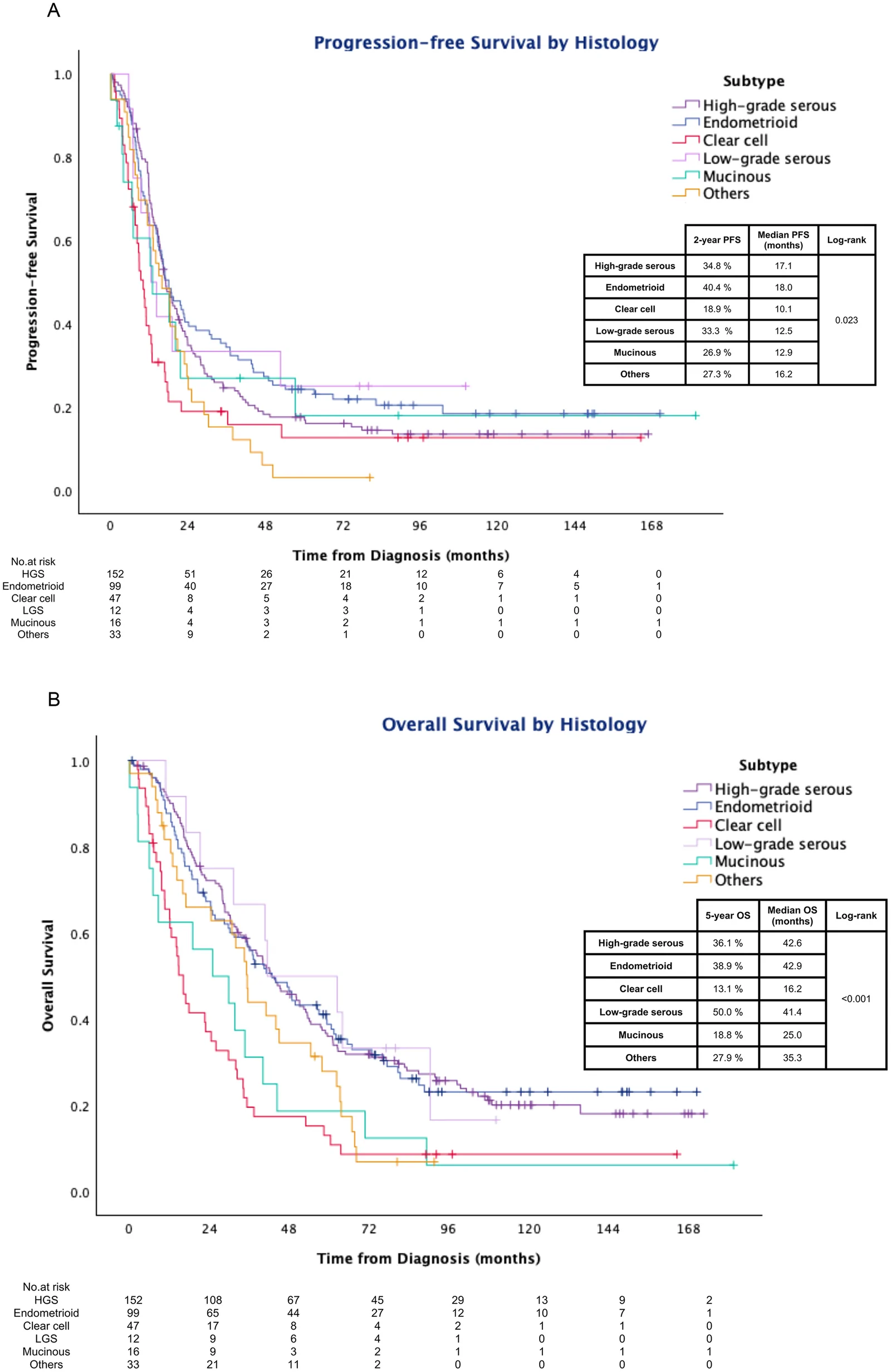
(A) Progression-free survival and (B) Overall survival by histological subtypes

**Figure 2:**
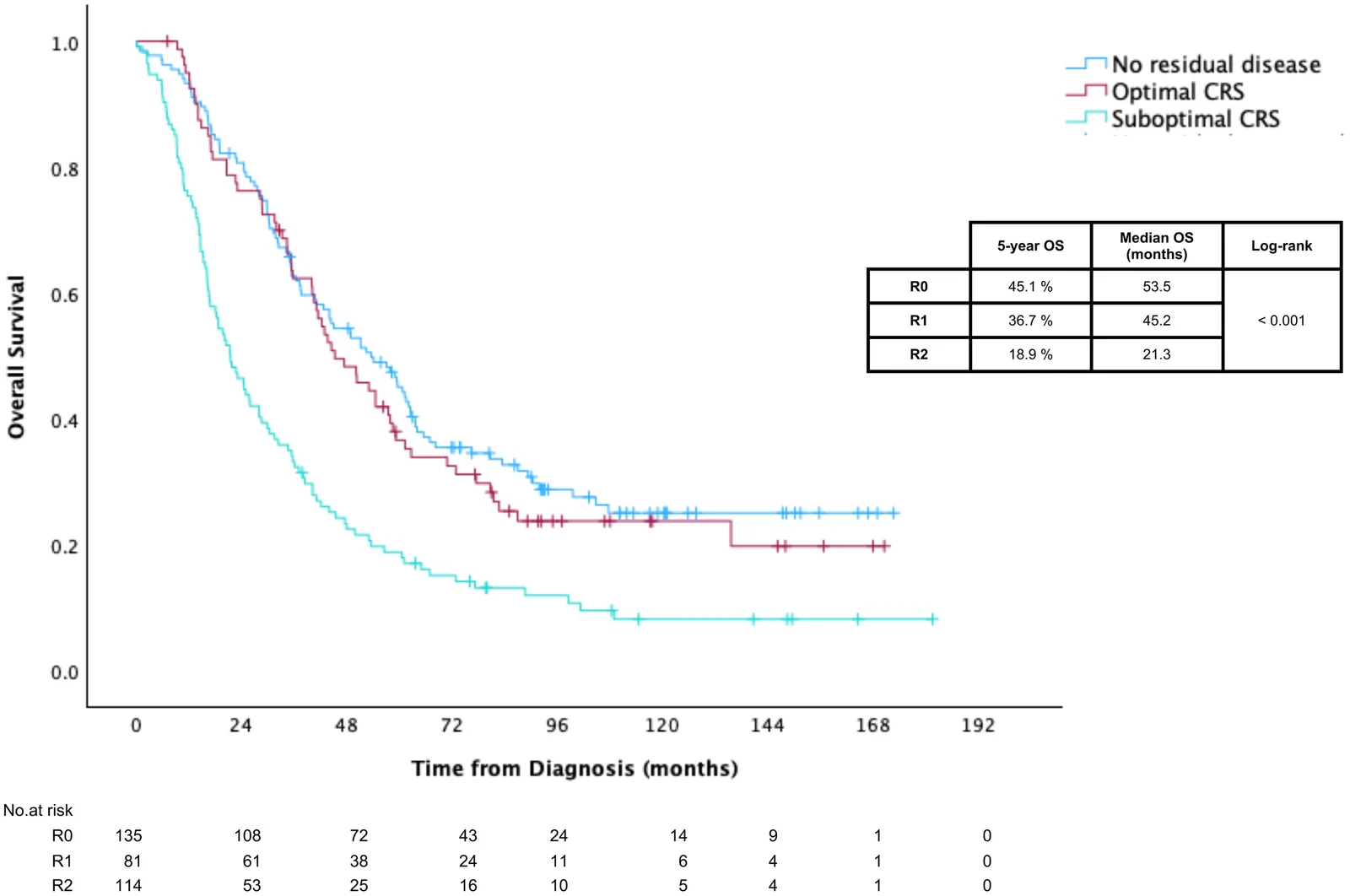
Overall survival by residual disease

**Figure 3:**
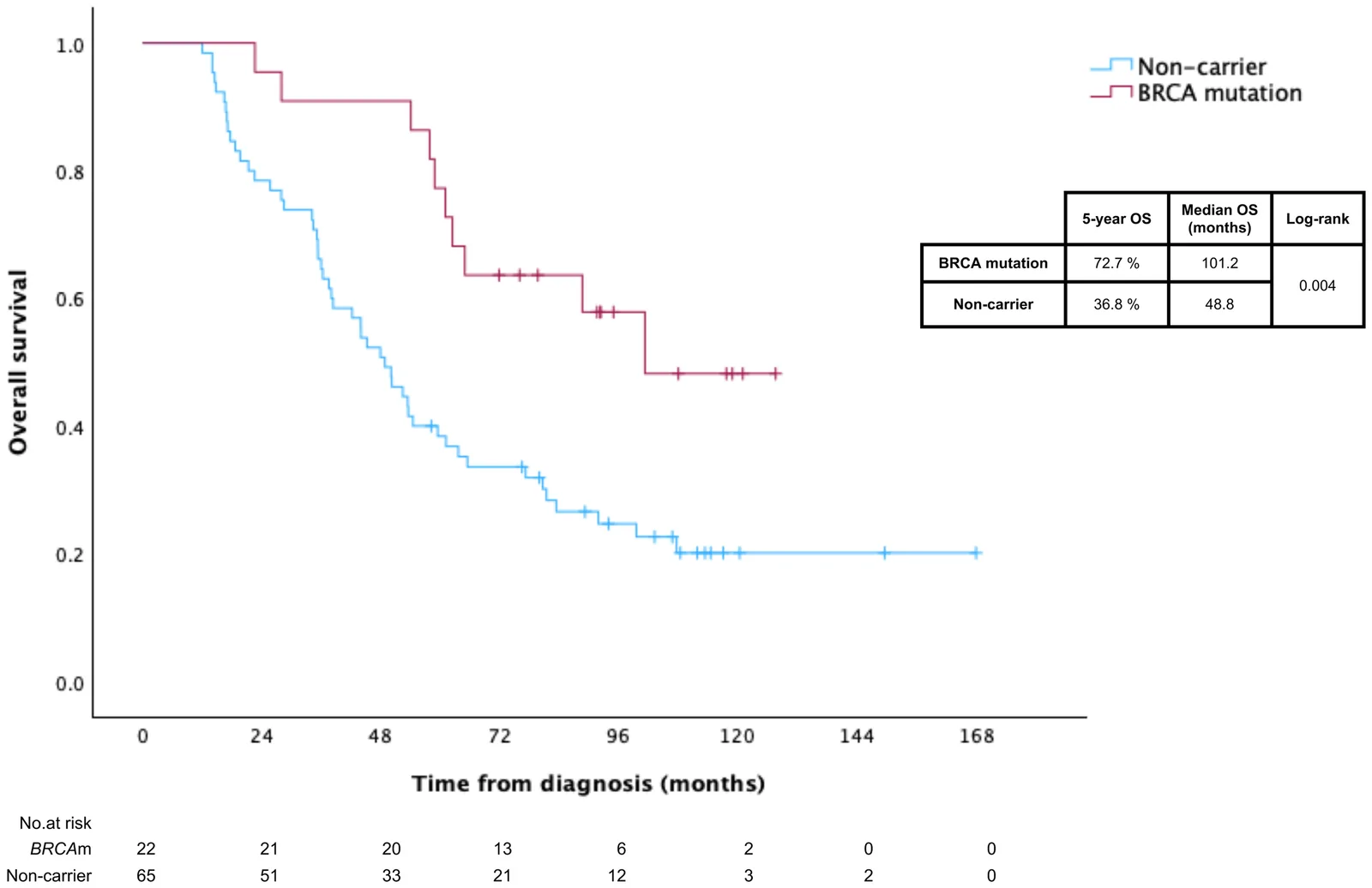
Overall survival by *BRCA* status

Kaplan-Meier curves and Cox proportional hazard analyses were performed to compare survival outcomes across histological subtypes (Table 4 and Figure 1). Advanced-stage clear cell carcinoma was strongly associated with inferior oncologic outcomes. The 5-year overall survival for clear cell carcinoma were 13.1%, with an approximately twofold increased risk of death compared with high-grade serous carcinoma (adjusted HR 2.01, 95% 1.37–2.95). Clear cell carcinoma was also associated with shorter progression-free survival, with a 2-year progression-free survival of 18.9% and an adjusted HR of 1.68. In contrast, the most common subtype, high-grade serous, had a 5-year overalls survival of 36.1% and 2-year progression-free survival of 34.8%. Low-grade serous and endometrioid carcinomas tended to demonstrate more favorable survival outcomes, whereas advanced-stage mucinous carcinoma, mixed histologic subtypes, and adenocarcinoma not otherwise specified tended to have poorer prognoses; however, these differences did not reach statistical significance. Residual disease was associated with a stepwise increase in both mortality and recurrence risk, with a twofold higher hazard for patients with residual disease ≥1 cm (Figure 2). Poor performance status was independently associated with decreased overall survival. *BRCA* mutation carriers had a significantly lower risk of mortality compared with noncarriers (adjusted HR 0.35, 95% CI 0.17–0.70), with corresponding 5-year overall survival of 72.7% vs 36.8%, respectively (Figure 3). Patients who did not undergo genetic testing also tended to have an increased risk of mortality, although this association did not reach statistical significance.

**Table 4:** Multivariable Cox proportional hazards analyses of progression-free and overall survival

	Progression-free survival		Overall survival	
	Adjusted HR (95% CI)	*P*-value	Adjusted HR (95% CI)	*P*-value
ECOG				.010
0–1	1 (reference)	.219	1 (reference)	
≥2	1.24 (0.88–1.75)		1.60 (1.12–2.29)	
BRCA status				<.001
No mutation	1 (reference)	.145	1 (reference)	
Mutation	0.69 (0.38–1.21)	.192	0.35 (0.17–0.70)	.003
Not tested	1.14 (0.82–1.56)	.439	1.28 (0.92–1.79)	.137
Stage				.880
III	1 (reference)	.733	1 (reference)	
IV	1.06 (0.77–1.46)		1.03 (0.74–1.43)	
Histology				
High-grade serous	1 (reference)	.004	1 (reference)	<.001
Endometrioid	0.75 (0.55–1.01)	.061	0.74 (0.54–1.03)	.075
Clear cell	1.68 (1.14–2.46)	.009	2.01 (1.37–2.95)	<.001
Low-grade serous	0.88 (0.44–1.73)	.701	0.86 (0.43–1.70)	.656
Mucinous	0.78 (0.41–1.52)	.470	1.28 (0.71–2.33)	.413
Others	1.27 (0.77–2.09)	.350	1.20 (0.70–2.03)	.508
Type of surgery		.299		.634
Primary cytoreductive surgery	1 (reference)		1 (reference)	
Interval debulking surgery	1.15 (0.88–1.50)		1.07 (0.81–1.41)	
Residual disease				
No residual disease	1 (reference)	<.001	1 (reference)	<.001
Residual <1 cm	1.07 (0.78–1.47)	.670	1.10 (0.79–1.53)	.572
Residual ≥1 cm	2.29 (1.72–3.06)	<.001	2.39 (1.78–3.20)	<.001

Manchana. Predictive factors for long-term survivors in advanced-stage epithelial ovarian cancer. Am J Obstet Gynecol 2026.

Manchana. Predictive factors for long-term survivors in advanced-stage epithelial ovarian cancer. Am J Obstet Gynecol 2026.

Manchana. Predictive factors for long-term survivors in advanced-stage epithelial ovarian cancer. Am J Obstet Gynecol 2026.

## Discussion

In this real-world cohort from a Southeast Asian tertiary center, approximately one-third of patients with advanced-stage epithelial ovarian cancer survived beyond 5-year, consistent with population-based daata from the Surveillance, Epidemiology, and End Results (SEER) database^9^ and higher than recent reposts from Western settings, likely reflecting the higher proportion of chemosensitive endometrioid histology in our cohort.^12^, ^13^, ^14^ The results indicate that long-term survival is influenced by multiple factors, including favorable tumor biology—specifically, *BRCA* mutation status and nonclear-cell histology—and the effectiveness of cytoreductive surgery. Importantly, the findings demonstrate an apparent prognostic disparity between clear cell carcinoma and high-grade serous carcinoma.

Advanced-stage ovarian clear cell carcinoma emerged as a strong negative prognostic factor. In this cohort, 13% of advanced-stage epithelial ovarian cancer cases were clear cell carcinoma, compared with 2.5% reported in the Gynecologic Cancer Intergroup (GCIG) meta-analysis,^15^ while in Japan, clear cell carcinoma accounts for approximately 25% of epithelial ovarian cancer,^16^ this result was consistent with our previous publication.^17^ Patients with clear cell carcinoma had substantially worse outcomes than those with high-grade serous carcinoma. Consistent with prior evidence,^15^^,^^18^^,^^19^ clear cell carcinoma may demonstrate favorable outcomes in early-stage disease; however, advanced-stage clear cell carcinoma in our cohort was associated with significantly inferior survival, primarily due to intrinsic resistance to standard platinum–taxane chemotherapy. Factors contributing to chemoresistance include alterations in oxidative stress response, hypoxia signaling (HIF-1α), angiogenesis, and glucose metabolism pathways.^20^

The strong association between *BRCA* mutation and 5-year survival observed in our cohort must be interpreted within its treatment context. Current evidence demonstrates that *BRCA* mutations confer their strongest survival benefit in the first 5 to 10 years after diagnosis, with hazard ratios for mortality crossing 1.0 at 4.8 years for *BRCA*1 and 10.5 years for *BRCA*2.^21^, ^22^, ^23^ Our 5-year endpoint is therefore optimally positioned to capture this early survival advantage. However, the survival difference between known *BRCA* carriers and noncarriers beyond the 5-year landmark did not reach statistical significance, consistent with observations by Kotsopoulos et al. that the *BRCA* survival advantage attenuates over extended follow-up.^24^ The introduction of PARP inhibitors has since transformed outcomes, with 7-year overall survival reaching 67% among *BRCA*-mutated patients receiving maintenance olaparib in the SOLO1 trial.^25^ Although only 9% of BRCA carriers in our cohort received PARP inhibitors due to limited access and reimbursement constraints, the association between *BRCA* mutation and 5-year survival underscores the intrinsic chemosensitivity of these tumors. Our findings provide a contemporary baseline and reaffirm that *BRCA* testing remains essential for prognostic stratification, particularly in resource-limited settings.

Suboptimal cytoreduction was independently associated with inferior survival, consistent with systematic reviews demonstrating a graded survival disadvantage with increasing residual disease burden.^26^ Real-world evidence from the SUROVA study further refines this paradigm, showing comparable overall survival between primary and interval debulking surgery, but a survival advantage for primary surgery confined to a highly selected subgroup achieving complete resection without postoperative complications, with treatment effects modified by ethnicity, including greater benefit from neoadjuvant treatment among Asian patients.^27^ In this cohort, there was a trend toward improved oncologic outcomes with primary cytoreductive surgery compared with interval surgery, although this did not reach statistical significance. These observations are conceptually aligned with the TRUST trial, which demonstrated that in nonfrail patients with resectable stage IIIB to IVB disease treated in high-volume expert centers, primary surgery achieved longer progression-free survival and numerically improved survival, with the most favorable outcomes observed in patients achieving complete cytoreduction.^28^ Although surgical morbidity was not evaluated in our study, the independent association between good performance status and long-term survival supports a risk-adapted treatment strategy that balances maximal cytoreduction against physiological reserve, surgical feasibility, and perioperative risk to optimize outcomes.

The low rate of genetic testing in our cohort reflects systemic barriers in many Asian healthcare systems, including cost constraints and limited access to genetic counseling.^29^^,^^30^ Similarly, the limited use of targeted therapies highlights persistent disparities in access to novel treatments in resource-limited settings.^31^^,^^32^ These findings highlight the urgent need to expand *BRCA* testing and PARP inhibitor reimbursement in Thailand and across the region. Future research should focus on developing effective treatments for chemoresistant histotypes, particularly clear cell carcinoma.

This study’s strengths include long-term follow-up, representation of diverse histologic subtypes reflecting real-world Southeast Asian practice, and contribution to the limited evidence base for this population. Several limitations warrant consideration. First, the retrospective design carriers inherent selection bias. Second, genetic testing was performed only a minority of patients, which may underestimate the true prevalence of *BRCA* mutations and introduce selection bias. Third, the use of a binary 5-year survival cutoff may reduce time-to-event information and introduce survivorship bias. Finally, the single-center design may limiting the generalizability of our findings to other populations and practice settings.

## Conclusion

Long-term survival in advanced epithelial ovarian cancer is strongly associated with *BRCA* mutation status, histology, residual disease, and performance status. Our 5-year analysis captures the critical early period when *BRCA* mutations exert their maximum survival advantage and when modern targeted therapies demonstrate their greatest impact. Suboptimal cytoreduction and high-risk histologies, particularly clear cell carcinoma, are associated with significantly worse outcomes. These findings emphasize the urgent need to expand access to genetic testing and PARP inhibitor therapy in resource-limited settings, where optimizing surgical management and identifying favorable tumor biology remain critical determinants of extended survival.

## CRediT authorship contribution statement

**Tarinee Manchana:** Writing – review & editing, Validation, Methodology, Data curation, Conceptualization. **Nicha Assavapokee:** Writing – original draft, Validation, Methodology, Formal analysis, Data curation, Conceptualization.

## Appendix Supplementary materials

Figure S1. Selection steps of study cohort Image, application 1

Table S1. Baseline Characteristics of Long-term Survivors by Recurrence Status Image, application 2

## Footnotes

Supplementary material associated with this article can be found in the online version at doi:10.1016/j.xagr.2026.100675.

## References

[1] Bray F., Ferlay J., Soerjomataram I., Siegel R.L., Torre L.A., Jemal A. (2018). Global cancer statistics 2018: GLOBOCAN estimates of incidence and mortality worldwide for 36 cancers in 185 countries. CA Cancer J Clin.

[2] Renz M., Friedlander M., Berek J.S. (2025). Cancer of the ovary, fallopian tube, and peritoneum: 2025 update. Int J Gynecol Obstetr.

[3] Howlader N., Noone A., Krapcho M. (2021). SEER cancer statistics review, 1975–2018. Natl Cancer Institute.

[4] Hoppenot C., Eckert M.A., Tienda S.M., Lengyel E. (2018). Who are the long-term survivors of high grade serous ovarian cancer?. Gynecol Oncol.

[5] Cress R.D., Chen Y.S., Morris C.R., Petersen M., Leiserowitz G.S. (2015). Characteristics of long-term survivors of epithelial ovarian cancer. Obstetr Gynecol.

[6] Akeson M., Jakobsen A.M., Zetterqvist B.M., Holmberg E., Brännström M., Horvath G. (2009). A population-based 5-year cohort study including all cases of epithelial ovarian cancer in western Sweden: 10-year survival and prognostic factors. Int J Gynecol Cancer.

[7] Kim S.J., Rosen B., Fan I. (2017). Epidemiologic factors that predict long-term survival following a diagnosis of epithelial ovarian cancer. Br J Cancer.

[8] Torre L.A., Trabert B., DeSantis C.E. (2018). Ovarian cancer statistics, 2018. CA Cancer J Clinic.

[9] Yang S.P., Su H.L., Chen X.B. (2021). Long-term survival among histological subtypes in advanced epithelial ovarian cancer: population-based study using the surveillance, epidemiology, and end result long-term survival database. JMIR Public Health Surveill.

[10] Zhou J., Wu S-G, Wang J. (2018). The effect of histological subtypes on outcomes of stage IV epithelial ovarian cancer. Front Oncol.

[11] Phung M.T., Pearce C.L., Meza R., Jeon J. (2023). Trends of ovarian cancer incidence by histotype and race/ethnicity in the United States 1992-2019. Cancer Res Commun.

[12] Shachar E., Raz Y., Rotkop G. (2025). Survivorship in advanced ovarian cancer: a prognostic model for overall survival and risk of recurrence. Oncologist.

[13] Bouchard-Fortier G., Panzarella T., Rosen B., Chapman W., Gien L.T. (2017). Endometrioid carcinoma of the ovary: outcomes compared to serous carcinoma after 10 years of follow-up. J Obstet Gynaecol Can.

[14] De Nonneville A., Zemmour C., Frank S. (2021). Clinicopathological characterization of a real-world multicenter cohort of endometrioid ovarian carcinoma: analysis of the French national ESME-Unicancer database. Gynecol Oncol.

[15] Mackay H.J., Brady M.F., Oza A.M. (2010). Prognostic relevance of uncommon ovarian histology in women with stage III/IV epithelial ovarian cancer. Int J Gynecol Cancer.

[16] Nagase S., Ohta T., Takahashi F., Enomoto T. (2019). Annual report of the committee on gynecologic oncology, the Japan Society of Obstetrics and Gynecology: annual patients report for 2015 and annual treatment report for 2010. J Obstet Gynaecol Res.

[17] Manchana T., Kobwitaya K. (2018). Survival outcomes for different subtypes of epithelial ovarian cancer. Clin Res Obstetr Gynecol.

[18] Anglesio M.S., Carey M.S., Köbel M., MacKay H., Huntsman D.G. (2011). Clear cell carcinoma of the ovary: a report from the first Ovarian Clear Cell Symposium, June 24th, 2010. Gynecol Oncol.

[19] Oliver K.E., Brady W.E., Birrer M. (2017). An evaluation of progression free survival and overall survival of ovarian cancer patients with clear cell carcinoma versus serous carcinoma treated with platinum therapy: an NRG Oncology/Gynecologic Oncology Group experience. Gynecol Oncol..

[20] Mabuchi S., Sugiyama T., Kimura T. (2016). Clear cell carcinoma of the ovary: molecular insights and future therapeutic perspectives. J Gynecol Oncol.

[21] Candido-dos-Reis F.J., Song H., Goode E.L. (2015). Germline mutation in BRCA1 or BRCA2 and ten-year survival for women diagnosed with epithelial ovarian cancer. Clin Cancer Res.

[22] McLaughlin J.R., Rosen B., Moody J. (2013). Long-term ovarian cancer survival associated with mutation in BRCA1 or BRCA2. J Natl Cancer Inst.

[23] Lavie O., Chetrit A., Novikov I., Sadetzki S., National Israeli Study of Ovarian Cancer (2019). Fifteen-year survival of invasive epithelial ovarian cancer in women with BRCA1/2 mutations: the National Israeli Study of Ovarian Cancer. Gynecol Oncol.

[24] Kotsopoulos J., Zamani N., Rosen B. (2022). Impact of germline mutations in cancer-predisposing genes on long-term survival in patients with epithelial ovarian cancer. Br J Cancer.

[25] DiSilvestro P., Banerjee S., Colombo N. (2023). Overall survival with maintenance olaparib at a 7-year follow-up in patients with newly diagnosed advanced ovarian cancer and a BRCA mutation: the SOLO1/GOG 3004 trial. J Clin Oncol.

[26] Bryant A., Hiu S., Kunonga P.T. (2022). Impact of residual disease as a prognostic factor for survival in women with advanced epithelial ovarian cancer after primary surgery. Cochrane Database Syst Rev.

[27] Chiva L., Ordas P., Mishra J. (2025). SUROVA study: global real-world treatment strategies and mortality risk prediction in advanced ovarian cancer. Int J Gynecol Cancer.

[28] Mahner S., Heitz F., Salehi S. (2025). TRUST: trial of radical upfront surgical therapy in advanced ovarian cancer (ENGOT ov33/AGO-OVAR OP7). J Clin Oncol.

[29] Kwong A., Tan DS-P, Ryu J.M., Consortium tA (2025). Current practices and challenges in genetic testing and counseling for women with breast and ovarian cancer in Asia. Asia-Pacific J Clin Oncol.

[30] Lin J., Feit J., Khoury A. (2021). Disparities in genetic testing for women with ovarian cancer: a systematic review. Gynecol Oncol.

[31] Bajwa A., Chidebe R.C.W., Adams T. (2025). Challenges and opportunities in ovarian cancer care: a qualitative study of clinician perspectives from 24 low- and middle-income countries. J Cancer Policy.

[32] Mizoguchi C., Okuma H.S., Muto M. (2024). Clinical landscape of precision oncology for rare cancers among diverse Asian populations: insights from the MASTER KEY registry. J Clin Oncol.

