# Supplementary material for: Predictive factors for long-term survivors in advanced-stage epithelial ovarian cancer
Source: AJOG Glob Rep. 2026 Jul 18;6(3):100675. doi: 10.1016/j.xagr.2026.100675 (PMC13495595; doi:10.1016/j.xagr.2026.100675)
Supplement: Supplementary file 1 [file mmc1.pdf]

Figure S1: Selection steps of study cohort

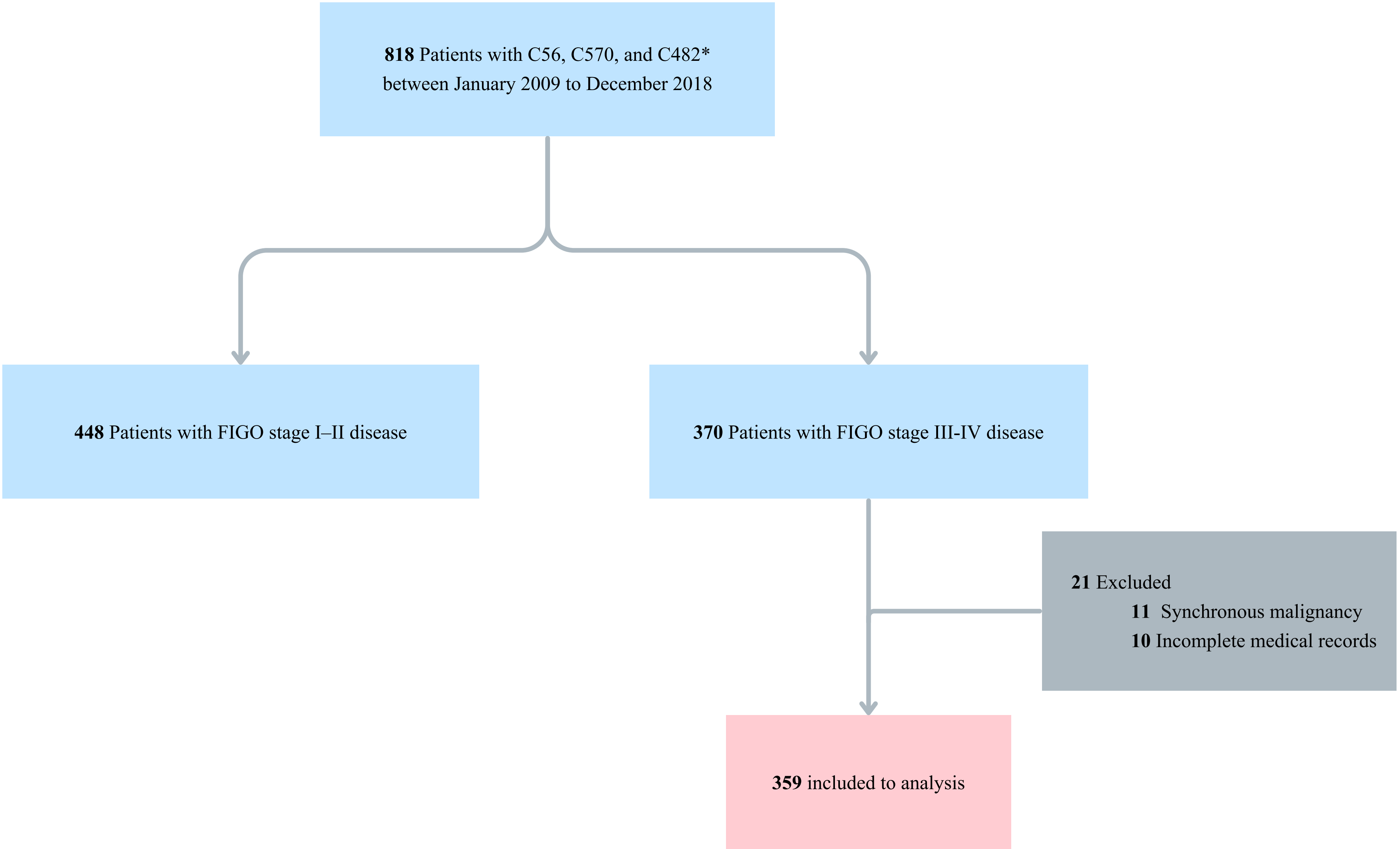

* ICD-10 Code - C56: Malignant ovarian carcinoma, C570: Carcinoma of fallopian tube, C482: Primary peritoneal carcinoma

Figure S2. Landmark Analysis of Overall Survival Among 5-Year Survivors with Known *BRCA* Status (N=41)

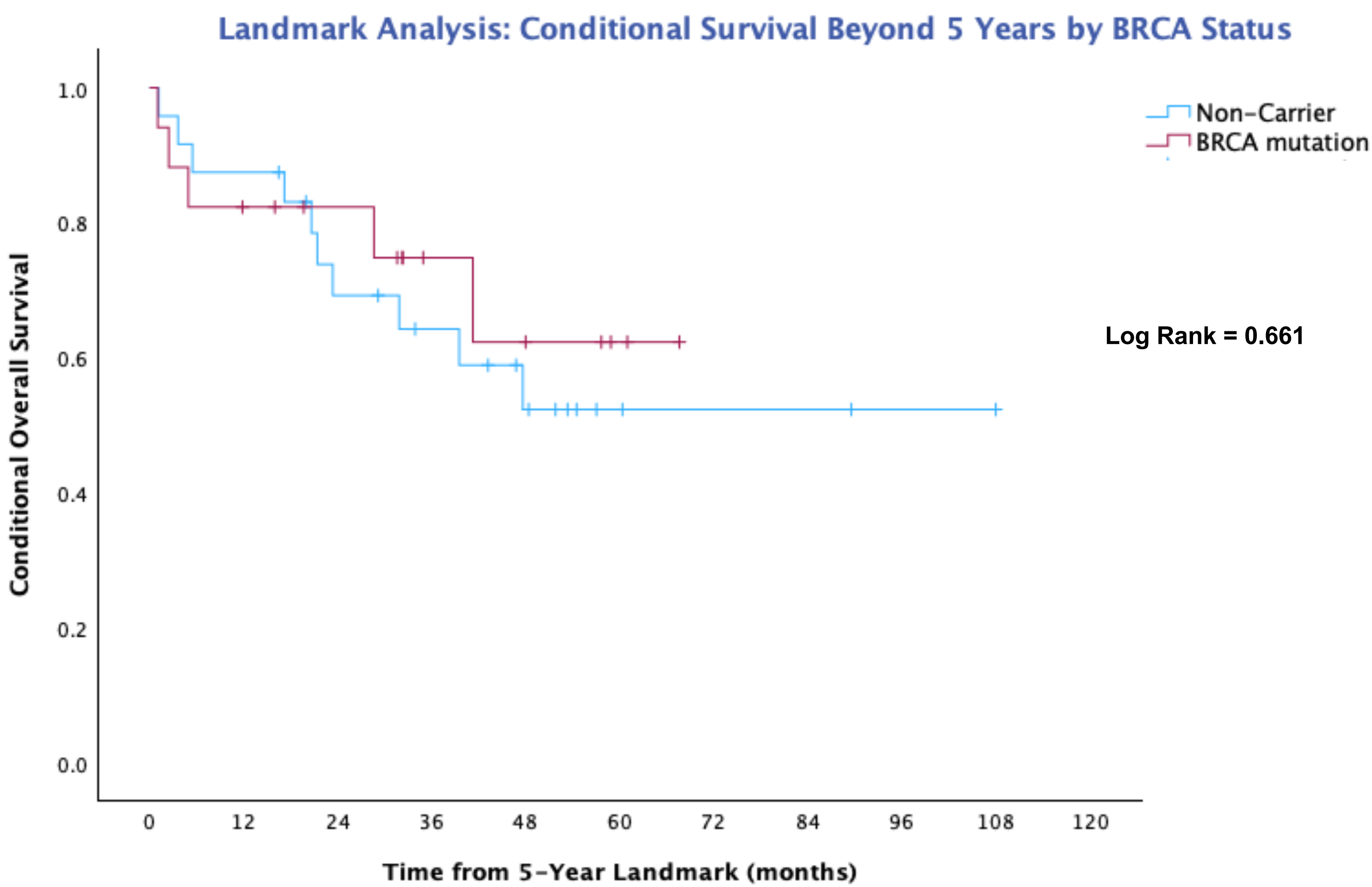

No.at risk						
Non-carrier	24	15	8	2	1	0
*BRCA*m	17	11	4	0	0	0
