# Supplementary material for: Predictive factors for long-term survivors in advanced-stage epithelial ovarian cancer
Source: AJOG Glob Rep. 2026 Jul 18;6(3):100675. doi: 10.1016/j.xagr.2026.100675 (PMC13495595; doi:10.1016/j.xagr.2026.100675)
Supplement: Supplementary file 2 [file mmc2.docx]

**Table** S1. Baseline Characteristics of Long-term Survivors by Recurrence Status (N=109)

|  | Non-Recurrence (n=45)  **n (%)** | Recurrence (n=64)  **n (%)** | **P-value** |
| --- | --- | --- | --- |
| **Age (years)** – Mean (SD) | *54.5 (11.1)* | *55.3 (10.3)* | 0.668 |
| ECOG  **0-1**  ≥ 2 | *41 (91.1)*  *4 (8.9)* | *56 (87.5)*  *8 (12.5)* | 0.758 |
| *BRCA* mutation  **No or unknown**  **Yes** | *40 (88.9)*  *5 (11.1)* | *52 (81.2)*  *12 (18.8)* | 0.279 |
| Primary Origin  **Ovary or Fallopian tube**  **Primary peritoneum** | *40 (88.9)*  *5 (11.1)* | *60 (83.7)*  *4 (6.3)* | 0.070 |
| FIGO Stage  **III**  **IV** | *38 (84.4)*  *7 (15.6)* | *51 (79.7)*  *13 (20.3)* | 0.528 |
| Histology  **High-grade serous**  **Endometrioid**  **Clear cell**  **Others** | *18 (40.0)*  *17 (37.8)*  *4 (8.9)*  *6 (13.3)* | *34 (53.1)*  *17 (26.6)*  *2 (3.1)*  *11 (17.2)* | 0.276 |
| Histologic grading  **Grade 1-2**  **Grade 3 or unknown** | *17 (37.7)*  *28 (62.3)* | *11 (17.2)*  *53 (82.8)* | 0.015 |
| Residual disease  **No residual**  **Residual < 1 cm.**  Residual ≥ 1 cm.  Not underwent surgery | *24 (53.3)*  *12 (26.7)*  *9 (20)*  *-* | *33 (55.0)*  *15 (25.0)*  *12 (20.0)*  *4 (6.3)* | 0.980 |
| Chemotherapy regimen  **Combined platinum-based**  Single **Carboplatin** | *44 (97.8)*  *1 (2.2)* | *58 (90.6)*  *6 (9.4)* | 0.236 |

**Table S2. Sensitivity Analysis: Multivariable Logistic Regression for 5-Year Long-Term Survival Among Patients with Known BRCA Status (N=87)**

|  | **Adjusted Odds Ratio** | **95% CI** | **P-value** |
| --- | --- | --- | --- |
| **ECOG**  0-1  ≥ 2 | 1 (reference)  0.62 | 0.54 – 7.08 | 0.697 |
| *BRCA* Status  No mutation  *BRCA* **mutation** | 1 (reference)  7.26 | 2.14 – 24.62 | 0.001 |
| Histology  High-grade serous  Endometrioid  Clear cell  Others | 1 (reference)  1.29  0.32  1.81 | 0.41 – 4.09  0.05 – 2.15  0.26 – 12.79 | 0.569  0.663  0.241  0.553 |
| Residual disease  No residual disease  Residual < 1 cm.  Residual ≥ 1 cm. | 1 (reference)  0.68  0.41 | 0.18 – 2.53  0.14 – 1.23 | 0.283  0.563  0.112 |
